# Analogous electronic states in graphene and planer metallic quantum dots

**DOI:** 10.1038/s41598-024-63465-2

**Published:** 2024-06-12

**Authors:** Ahmed M. Othman, Mohammad A. Kher-Elden, Fatma Ibraheem, Moukhtar A. Hassan, Mohammed Farouk, Zakaria M. Abd El-Fattah

**Affiliations:** 1 Physics Department, Faculty of Science, Al-Azhar University, Nasr City, Cairo, 11884 Egypt; 2 Physics Department, Faculty of Science, Al-Azhar University Girls Branch, Nasr City, Cairo, 11753 Egypt; 3 Physics Department, Faculty of Science, Galala University, New Galala City, Suez, 43511 Egypt

**Keywords:** Quantum physics, Physics, Condensed-matter physics, Surfaces, interfaces and thin films

## Abstract

Graphene nanostructures offer wide range of applications due to their distinguished and tunable electronic properties. Recently, atomic and molecular graphene were modeled following simple free-electron scattering by periodic muffin tin potential leading to remarkable agreement with density functional theory. Here we extend the analogy of the $\pi$-electronic structures and quantum effects between atomic graphene quantum dots (QDs) and homogeneous planer metallic counterparts of similar size and shape. Specifically, we show that at high binding energies, below the $\overline{M}$-point gap, graphene QDs enclose confined states and standing wave quasiparticle interference patterns analogous to those reported on coinage metal surfaces for nanoscale confining structures such as vacancy islands and quantum corrals. These confined and quantum corral-like states in graphene QDs can be resolved in tomography experiments using angle-resolved photoemission spectroscopy. Likewise, the shape of near-Fermi frontier orbitals in graphene quantum dots can be reproduced from electron confinement within homogeneous metal QDs of identical size and shape. Furthermore, confined states analogous to those found in metallic quantum stadiums can be realized in coupled QDs of graphene for reduced separation. The present study offer a simple fundamental understanding of graphene electronic structures and also open the way towards efficient modeling of novel graphene-based nanostructures.

## Introduction

The atomically-thick honeycomb carbon lattice, known as graphene, is an intriguing two-dimensional (2D) model system with distinguished relativistic electronic properties and potential technological applications^1–5^. While the gapless electronic band structure of free-standing extended graphene is appealing for applications such as transport^3,5–7^ and plasmonics^8,9^, the absence of a natural energy gap limits its integration in various nanoelectronic devices. Several routes are envisioned to induce energy gaps, including the symmetry breaking of the carbon-sublattices^10,11^ as well as the fabrication of graphene nanostructures, such as edge-controlled nanoribbons^5,12–14^ and quantum dots (QDs)^2,15–17^, where quantum confinement leads to tunable band gaps.

The growing interest in the unique physics and applications of graphene and its nanostructures has stimulated the search for graphene-like artificial materials with tunable lattice parameters. In this context, surface electrons confined into honeycomb pathways by on-surface repulsive CO potentials, engineered by scanning tunneling microscopy (STM) tip manipulation, allowed the fabrication of artificial molecular graphene, in which several graphene characteristics such as Dirac point and strain induced pseudo-magnetic field were successfully realized^18–20^. Moreover, the same approach, i.e., the confinement of metallic free electron into honeycomb pathways, has been utilized later to model the electronic structure of atomistic graphene to density functional theory (DFT) level of accuracy using the scattering potential as a single fitting parameter^21^. This simplified nearly free electron approach remains valid for graphene nanostructures^21^, carbon-based organic molecules^22^, and other 2D materials such as hexagonal boron nitride^23^. Similarly, the discretization of the electronic structure in small graphene QDs, such as molecular coronone, could be well reproduced from confined electrons in smooth and/or atomically corrugated quantum wells, where the spectral intensity as probed with angle resolved photoemission (ARPES) as well as the frontier orbitals coincides with DFT calculations^24^. In fact, the electronic band structure of graphene is comparable to that of a 2D homogeneous metal, except for the large $\overline{M}$-point gap induced by the atomic potential, and thereby they should share similar physics at some aspects. The distinction is primarily for the near-Fermi electrons, where triangular electron and hole pockets around the $\overline{K}$-points and the associated intravalley scattering are solely present for graphene structures^21,25–28^.

Here we make use of the electron plane wave expansion (EPWE) method to quantitatively establish a solid analogy between the electronic properties of graphene and lateral 2D metallic QDs. We show that relatively small graphene QDs exhibit electronic structures, confined states, and quasiparticle standing wave patterns analogous to those reported for confining metallic nanostructures such as vacancy islands^29,30^, molecular networks^31,32^, and quantum corrals^33–35^. Additionally, the wavefunction morphology of the frontier orbitals in graphene QDs, although present at the energy window of the $\overline{M}$-point gap, can be reproduced from confinement inside homogeneous metallic QDs. We further demonstrate that this comparison remains valid even for more complex nanostructures such as coupled graphene QDs and elliptical quantum stadium. For large nanoscale graphene QDs, however, graphene distinguished electronic confined states and peculiar Friedel oscillations^36–38^ resulting from confined/scattered Dirac electrons with intravalley scattering wavevector finds no correspondence in metallic QDs. In the limit where such intravalley scattering is not relevant, the present study allows the simplification of the underlying physics of graphene by a comparative analysis of the corresponding homogeneous 2D metal, thereby offering an efficient modelling technique for diverse carbon-based nanostructures.

**Figure 1:**
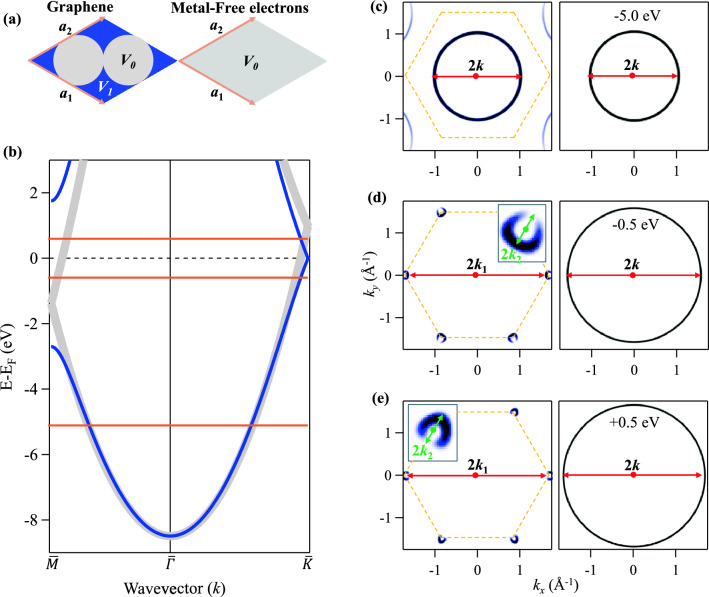
Comparative electronic properties of graphene and 2D metal. (**a**) The 2D hexagonal unit cell with lattice parameters $a_1$ = $a_2$ = $\sqrt{3}$ *a*, where *a* = 1.42  Å is the carbon-carbon distance, and the predefined potential landscapes utilized in EPWE calculations for graphene (left) and 2D homogeneous metal (right). The gray circles defining the two carbon atoms, each of radius *R* = 0.71  Å  are filled with a potential $V_0$ = 0 eV, while the blue interstitial region stands for the carbon voids with a strong confining potential of $V_1$ = 22.95 eV. Within the metal unit cell a homogeneous potential (gray) $V_0$ = 0 is set everywhere. (**b**) Calculated band structure using EPWE along $\overline{\Gamma M}$ (left) and $\overline{\Gamma K}$ (right) directions within the first Brillouin zone (BZ) for graphene (blue) and metal (gray). Closed gaps are evident for both metal and graphene at the high symmetry $\overline{K}$-point, while an energy gap opens up, solely for graphene, at the $\overline{M}$-point. (**c**–**e**) Simulated constant energy surfaces (CESs) taken at the energies indicated by the orange lines in (**b**), i.e., at − 5.0 eV (**c**), − 0.5 eV (**d**), and + 0.5 eV (**e**) for graphene (left) and metal (right). The insets in (**d**, **e**, left) highlight the triangular wrapping of the graphene pockets.The red arrows denote the diameters of the CESs (2*k*) and intervalley scattering wavevector in graphene (2$k_1$), while green arrows correspond to intravalley scattering wavevectors (2$k_2$). The dashed hexagons in (**c**–**e**) correspond to graphene BZ .

## Results and discussion

### Electronic properties of extended graphene vs. 2D metal

Figure 1 presents the one-to-one correspondence between the electronic structure of extended graphene and a pristine 2D homogeneous metal as obtained from EPWE calculations. The potential landscape corresponding to the hexagonal graphene unit cell, defined by the lattice vectors $a_1$ = $a_2$ = $\sqrt{3}$ *a* (*a* = 1.42  Å being the carbon-carbon distance), is depicted in Fig. 1(a, left). It contains two carbon atoms each of radius *R* = 0.71 Å enclosing a muffin tin potential $V_0$ = 0 (gray) embedded in a region of high confining potential $V_1$ = 22.95 eV (blue)^21^. The same unit cell parameters are used for the 2D metal where $V_0$ and $V_1$ are both set to zero defining an empty metallic lattice, as shown in Fig. 1(a, right). The effective mass for graphene is chosen to be $m_{\text {eff}}$ = 1 $m_e$, i.e., the rest mass of electrons, and the reference energy is adjusted to locate the Fermi energy $E_F$ at the Dirac point. For a proper comparison with the hypothetical metal with free electron parabolic dispersion, the effective mass was finely tuned to $m_{\text {eff}}$ = 1.165 $m_e$ and the reference energy is chosen to obtain the same band minima, i.e., $\overline{\Gamma }$-point energy, for graphene and metal. Using the potential landscapes in Fig. 1a and the so defined scattering parameters, the EPWE electronic band structures of graphene (blue) and homogeneous 2D metal (gray) are depicted in Fig. 1b.

Both graphene and the metallic system exhibit practically identical free-electron dispersions in the energy window spanning from the common $\overline{\Gamma }$-point energy, at $\sim -$ 8.365 eV, up to $\sim -$ 4.0 eV. At higher energies towards the Fermi energy, both systems maintain similar dispersion along the $\overline{\Gamma K}$ direction up to the graphene Dirac point located at $E_F$. Along the $\overline{\Gamma M}$ direction, however, a $\overline{M}$-point band gap opens up solely for graphene, with the lower and upper edges positioned at $\sim -$ 2.49 eV and $\sim$ 1.84 eV, respectively. Such a distinct difference between atomic graphene and homogeneous metal should lead to radical transformation of the otherwise circular constant energy surfaces (CESs), taken at/nearby the gap energies as we show next.

In Fig. 1c–e we present the simulated photoemission intensity of the constant energy surfaces taken at selected energies for graphene (left) and the 2D metal (right). At an energy of − 5.0 eV (c), where the band dispersion of the two systems coincide, both graphene and the 2D metal exhibit circular CESs of identical diameter (2*k*) enclosed within the Brillouin zone (BZ) defined by the dashed hexagon, although additional umklapp contours can be revealed at higher BZs for graphene. In fact, these circular CESs of identical diameters exist for all electron energies spanning from $\overline{\Gamma }$-point energy up to $\sim -$ 4.0 eV, as shown in the movies S1 and S2. At higher energies, the CESs of the metal keep its circular shape with increasing diameters (d, e, right), while those of graphene undergo additional progressive deformation (see movie S2), and eventually transform into highly featured and gapped CESs at energies close to the Dirac point (d, e, left). The CESs of graphene at energies slightly below (− 0.5 eV) and above (+ 0.5 eV) the Fermi energy consist of the six typical triangular pockets centered around the $\overline{K}$-points^21,28^. The triangular wrapping of these pockets, see insets in (d, e, left), which originates from deviation from the linear dispersion near the $\overline{K}$-point and electron-hole asymmetry^3,39^, demonstrates the suitably of our EPWE approach to correctly reproduce the electronic structure of graphene to DFT level of accuracy^21^, which would require a second-nearest-neighbor hopping to be captured by tight binding models. Furthermore, the intensity distribution within each pocket is non-uniform, exhibiting significant variations when traversing around the $\overline{K}$-points, which notably assumes the role of pseudospin in shaping the dispersion of the Dirac cone^28^.

The comparative CESs presented in Fig. 1c–e offer valuable information about the distinct electron scattering in graphene and metallic nanostructures, such as quantum dots. For instance, at − 5.0 eV the circular CESs of identical diameter (c), assures that the confining properties are similar and the quasiparticle oscillations from graphene and metallic QDs of the same size and shape, and/or due to scattering by defects, should host the same type and period of oscillation as given by the scattering wavevector 2*k*^26,40–43^, analogous to the standing wave patterns recurrently reported for metallic quantum corrals^33,35,44^. Quantitatively, however, the CESs of graphene additionally display subtle umklapp intensity attributed to reflections from adjacent BZs, which are expected to have minimal contribution to the scattering. We, therefore, anticipat that QDs made of graphene should host quasiparticle interference patterns and analogous quantum corrals states for electron energies in the range $\sim -$ 8.365 eV to $\sim -$ 4.0 eV as we demonstrate later. At energies near the $\overline{K}$-point (d, e), however, the CESs of metal maintain their circular shape with increasing diameters, i.e., scattering wavevector 2*k*, while the featured and gapped CESs of graphene afford two distinct scattering wavevectors 2$k_1$ and 2$k_2$, namely the intervalley and intravalley quasiparticle scattering, as denoted by the red and green arrows, respectively^3,26,40,45^. Intervalley scattering between states from adjacent triangular pockets should afford similar period of oscillation ($2k_1$) as the pristine metal, while intravalley scattering within each pocket, beside the need for pseudospin flip^3,40,45,46^ and its sensitivity to the direction of propagation with respect to the graphene lattice^25^, provides much longer oscillations given the corresponding small pocket diameter ($2k_2$), and thereby requires sufficiently large nanometer scale graphene quantum dots for the identification of their nanoscale quantum interference patterns^36,37,40,45^.

The above analysis and discussion revealed a strong correspondence for the confinement and scattering properties in graphene and 2D homogeneous metallic nanostructures at electron energies below the lower edge of the $\overline{M}$-point gap. This analogy can even be extended up to the Fermi energy for a relatively small confining nanostructures, as long as the scattering in graphene nanostructures is dominated by intervalley scattering. In the following we opt up for quantitative demonstration of such a remarkable analogy between graphene and metallic quantum dots (GQDs and MQDs) for small and moderate QD sizes.

**Figure 2:**
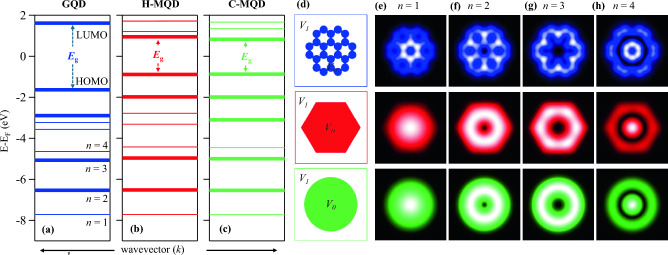
Graphene vs. metallic quantum dots: the confined states. (**a**–**c**) The EPWE calculated electronic structure for (**a**) the smallest graphene quantum dot (GQD) and (**b**, **c**) for the same size 2D metallic quantum dots (H-MQG and C-MQD) of hexagonal and circular shapes, respectively, with their potential landscapes depicted in (**d**). In the three cases, the electronic structure consists of energy levels and near-Fermi gap defining the frontier highest occupied (HOMO) and lowest unoccupied (LUMO) molecular orbitals. (e-h) Local density of states (LDOS) simulations at the energy of the first four energy levels *n* = 1–4 for GQD (top), H-MQD (middle), and C-MQD (bottom). The progressive transformation from dome-, donut-, to sombrero-like shape is demonstrated for all QDs.

### Graphene and metallic quantum dots: confined states

Figure 2 illustrates the remarkable similarity between the electronic characteristics of the smallest GQD, namely coronene, and the lateral MQDs of identical surface area but possessing circular (C-MQD) and hexagonal (H-MQD) shapes. The EPWE potential landscapes for these quantum dots are shown in Fig. 2d, where $$V_0$$ = 0 and $$V_1$$ = 22.95 eV, and the corresponding calculated electronic structures are depicted in Fig. 2a–c. In Fig. 2d, the GQD constitutes 24 carbon atoms (blue) each has the same diameter (1.42 Å) as used for extended graphene. The metallic quantum dots, however, are modeled as homogenous regions of either hexagonal (middle) or circular (bottom) shape. The electronic structure of GQD in Fig. 2a consists of discrete energy levels sliced from the graphene’s valence and conduction bands each contributing 12 energy levels separated by a HOMO-LUMO energy gap ($$E_g$$ $$\sim$$ 2.7 eV), where HOMO and LUMO refer to the frontier highest occupied and lowest unoccupied molecular orbitals, respectively, in agreement with DFT calculations^47,48^. Similar band structures are obtained for the H-MQD (b) and C-MQD (c) both exhibiting smaller energy gap ($$E_g$$ $$\sim$$ 1.8 eV) compared to GQD, due to the absence of atomic potential corrugations within the homogeneous interior of these metallic QDs. However, we can readily observe that the energetic positions of the first four energy levels *n* = 1–4 and their degeneracy, expressed here as thicker levels, are coincident with those of GQD, i.e., non-degenerate *n* = 1, 4 and degenerate *n* = 2, 3 states. Additionally, except for the energetic position, the number of levels and their degeneracy up to and including the frontier HOMO and LUMO levels are reproduced for graphene and metallic QDs. For the GQD and H-MQD, however, the degeneracy of the *n*=5 state is lifted compared to C-MQD, indicating that this particular electronic state in graphene and metallic QDs is sensitive to the wrapping and precise shape of the confining nanostructures. The similarities between GQDs and MQDs are further highlighted by comparing the wavefunctions ($$\psi$$) corresponding to each energy level. Fig. 2e–h present a comparative EPWE calculated $$|\psi ($$x, y$$)|^2$$, i.e., the local density of states (LDOS), for GQD (top), H-MQD (middle), and C-MQD (bottom), taken at the energies of *n* = 1–4 states. The non-degenerate *n* = 1 state exhibits the typical dome-like shape with maximum intensity at the QD center for the three types of QDs, although for GQD the QD center refers to the central benzene ring, not the carbon-free central void^2^. For the degenerate *n* = 2 (f) and *n* = 3 (g) states, the intensity is depleted at the QD center where the probability density takes a donut-like form of similar wrapping as the QD shape and with progressively increasing diameter towards the QD boundaries for *n* = 3 state^2^. At the energy of *n*=4 level (h), the LDOS revealed strong intensity back at the center with additional weaker intensity contribution at the QD’s termini, thereby taking a sombrero-like shape^49,50^. Overall, it is clear that the full electronic band structure, particularly below $$\sim -$$ 4.0 eV, and the simulated LDOS of the confined states in GQDs can be well simplified by assuming electron confinement inside homogeneous metallic QDs of the same size and shape, where here the H-MQDs offer better matching with the electronic characteristics observed in GQD.

**Figure 3:**
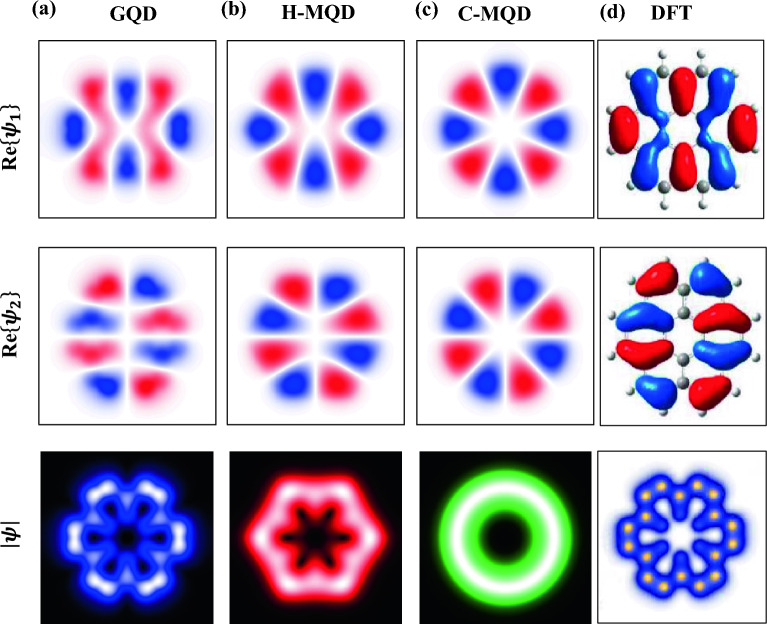
Graphene vs. metallic quantum dots: the HOMO states. The EPWE calculated real part of the wavefunctions Re$\{\psi _1$} (top) and Re$\{\psi _2$} (middle) corresponding to the degenerate HOMO and their resulting convoluted LDOS $|\psi|^2$| (bottom) for coronene GQD (**a**) as well as hexagonal (**b**) and circular (**c**) MQDs compared to DFT calculations (**d**) adapted from Refs.^47,48^. The disclosed detailed oscillations in the electron probability distribution including the nodal and antinodal features are practically identical for EPWE calculated GQD and H-MQD with DFT calculations.

In order to trace the extent of this obvious analogy between graphene and metallic QDs we explore the near-Fermi electronic states, such as the HOMO, where at such energies the band structure and CESs of graphene are distinct from pristine metal as demonstrated in Fig. 1b, d, e. The HOMO of GQD is positioned at $\sim -$ 1.8 eV, i.e., inside the $\overline{M}$-point gap of extended graphene, where the CES at such energy allows for both intervalley and intravalley scattering, Fig. 1d, e. For the smallest GQD here explored we anticipate minimal contribution due to intravalley scattering, and electron scattering in such a small graphene nanostructures is dominated by intervalley scattering, and thereby remains comparable to pristine metal nanostructures. Our EPWE simulations for the HOMO, presented in Fig. 3, clearly affirm these arguments. In Fig. 3 the real part of the simulated wavefunctions Re$\{\psi _1$} and Re$\{\psi _2$} for the doubly degenerate HOMO are presented together with the convoluted LDOS $|\psi|^2$| for the coronene QD (a), and for the hexagonal (H-MQD) (b) and circular (C-MQD) (c) metallic QDs. These are compared to the DFT calculations depicted in Fig. 3d for coronene, as extracted from Refs.^47,48^. First, we can see a decent agreement between the orbitals shape of coronene obtained by our EPWE approach (a) and $ab-inito$ DFT calculations (d), reassuring the suitability of EPWE for the exploration of finite GQDs^21,22^. Most importantly, the orbital shapes resemble those obtained for the metallic quantum dots (b, c), where the hexagonal MQD revealed excellent matching with EPWE and DFT calculations for coronene. This finding is in agreement with earlier work performed for coronene and hexabenzocoronene^24,47,48,51^, and further reaffirms that the confinement within GQDs is dominated by the size effect, i.e., scattering at the boundaries, rather than the atomic potential corrugation, and therefore can be simply understood in terms of free electrons confined to homogeneous metallic nanostructures of identical size and shape.

**Figure 4:**
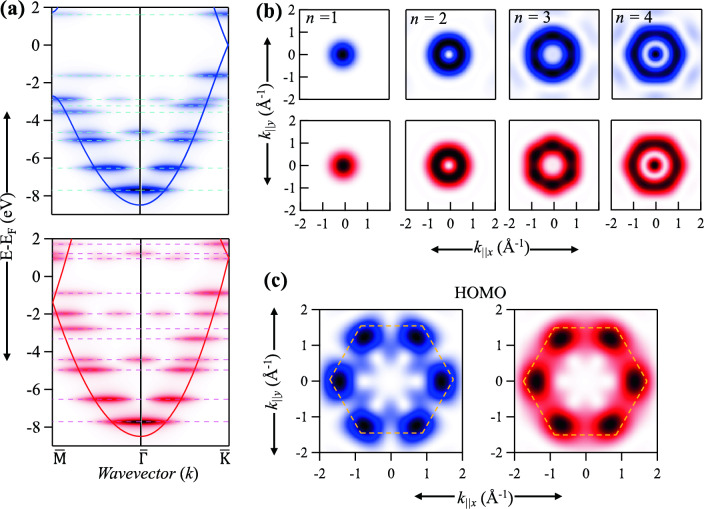
EPWE-simulated ARPES data for graphene and metallic quantum dots. (**a**) Simulated photoemission intensity along the $\overline{\Gamma M}$ (left) and $\overline{\Gamma K}$ (right) directions for coronene GQD (top) and H-MQD (bottom). The band structures of the corresponding extended systems (solid curves) and energy levels of the QDs (dashed lines) are superimposed. (**b**) Comparative simulated CESs for the first four levels *n* = 1–4 of coronene (top) and H-MQD (bottom). (**c**) Simulated CESs corresponding to the HOMO of coronene (left) and H-MQD (right), both revealing intensity at/near the corners of graphene’s BZ (dashed hexagon).

While the HOMO energy and wavefunction in such a small GQD can and has been already probed experimentally by scanning tunneling microscopy/spectroscopy (STM/STS)^52^, the *n* = 1–4 confined states in Fig. 2 are not accessible, due to their high binding energies that demand high bias voltages which affects the integrity of the STM tip. However, they can be readily probed using suitable techniques such as angle resolved photoemission spectroscopy (ARPES) combined with Fourier transform and phase recovery algorithms as demonstrated in the tomographic reconstruction of the orbital shape for several planar molecules from ARPES data^53–55^. In Fig. 4 we provide comparative simulations of the photoemission dispersion and selected constant energy surfaces (CESs) for coronene, i.e., GQD, and for the H-MQD. Consistent with our analysis in Figs. 2, 3, the photoemission intensity (a) for the GQD (top) and H-MQD (bottom), calculated along $\overline{\Gamma M}$ (left) $\overline{\Gamma K}$ (right) directions, are similar each consisting of a sequence of discrete energy levels (dashed lines) with energy and wavevector modulated intensity. The main intensity is localized near the band structure curves of the corresponding extended systems (solid lines) with some intensity modulation due to quantization along the $\overline{M \Gamma K}$ directions, including antinodes at the center of the BZ ($\overline{\Gamma }$-point), in agreement with earlier measurements and calculations for coronene^24,51^. To unveil a comprehensive perspective of the system’s electronic structure, we exploit the complete $k_x$–$k_y$ dependent intensity distribution, i.e., the CESs, for selected confined states, thus extracting additional insights from these intricate intensity patterns which after suitable Fourier transform routines yields the corresponding orbital shapes^53–55^. In Fig. 4b the simulated CESs corresponding to the first four energy levels *n* = 1–4 are depicted for GQD (top) and H-MQD (bottom). The two sets of CESs are practically identical for the two types of QDs, although for GQD weak photoemission intensity at high $k_x$-$k_y$ values originating at the neighboring BZs is present particularly for *n* = 3, 4 states. The shape of these CESs resemble the corresponding real-space LDOS of the confined states in Fig. 2e–h, i.e., they transform from the dome-like shape for *n* = 1 CES, to donut-like shapes with hexagonal warping for *n* = 2, 3, and eventually to the sombrero-like topology at *n* = 4. For GQDs grown onto metallic or semiconductor substrates these confined state can be identified by ARPES, and the corresponding real-space LDOS, in Fig. 2e–h, can be deduced from the tomographic reconstruction following typical Fourier transform algorithms^56–59^. Likewise, the simulated CESs corresponding to the HOMO are depicted in Fig. 4c for GQD (left) and H-MQD (right), both exhibiting hexagonal symmetry with photoemission intensity maximized near/at the $\overline{K}$-points of graphene BZ (dashed hexagon)^28^. The topology of these CESs agreed remarkably well with DFT calculations and with ARPES measurements conducted on coronene^24^. Therefore, for such a small GQD, the entirely electronic band structure as well as the real-space morphology of confined states and frontier orbitals can be nicely reproduced by exploring the equivalent homogeneous metallic QDs, and this GQD-MQD correspondence can be experimentally verified using ARPES, i.e., by measuring the absolute value of the initial-state wavefunction in reciprocal space (CES) and subsequent Fourier transformation to enable the reconstruction of molecular orbital densities in real space^53^.

### Moderate size graphene quantum dots: quantum corrals

When free electrons are spatially restricted within length scales near the de Broglie wavelength, their behavior becomes predominantly influenced by quantum mechanical phenomena. Consequently, the construction of atomic-scale barriers designed to confine these electrons, such as *quantum corrals*, results in the formation of electron standing waves through the interference of incident and reflected waves. These spatial oscillations represent quantum interference patterns arising from the scattering of confined electrons at the barriers^33,44^. In the following we demonstrate the analogous occurrence of the quantum corral standing wave interference patterns, recurrently reported for pristine noble metals^33–35,44,60^, on moderate size GQDs. Here we are primarily interested in the $\pi$-band electrons near the $\overline{\Gamma }$-point, i.e., below $\sim -$ 4.0 eV, although near-Fermi Dirac electrons have shown to exhibit behavior akin to Friedel oscillations in large nanoscale size GQDs^37,61^.

**Figure 5:**
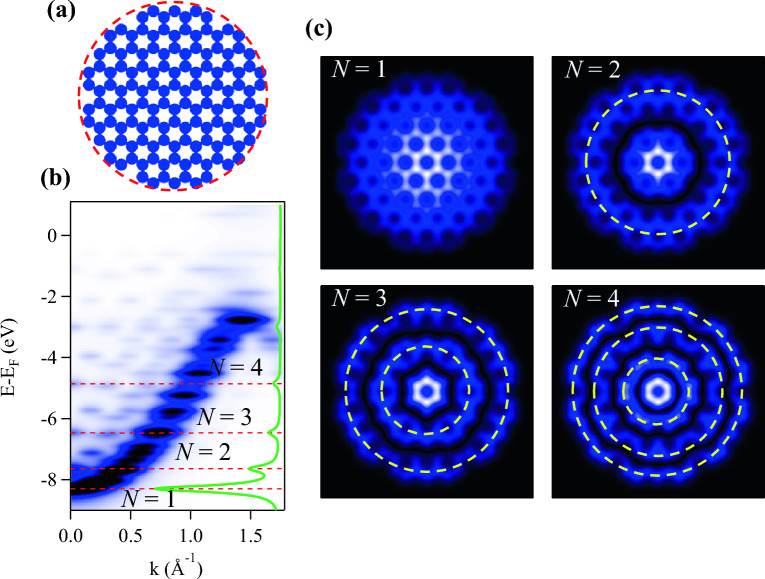
Graphene quantum corrals. (**a**) EPWE potential landscape for a moderate size (*r* $\sim$ 1 nm) semi-circular GQD. (**b**) Simulated ARPES dispersion along the $\overline{\Gamma M}$ direction. The green spectrum corresponds to a line profile taken at the $\overline{\Gamma }$-point demonstrating the first four levels (*N* = 1–4) with central antinodes at $\overline{\Gamma }$, see dashed red lines. (**c**) LDOS simulations for these *N* = 1–4 states showing the formation of standing waves with progressively increasing number of antinodal rings.

Figure 5a depicts the EPWE potential landscape for a moderate size (*r* $\sim$ 1 nm) GQD of a nearly circular shape. The large number of contained carbon atoms leads to a rich electronic structure consisting of numerous energy levels, with strong photoemission intensity modulation, mainly maximized near the graphene band, as presented in Fig. 5b for ARPES simulation along the $\overline{\Gamma M}$ direction. A line profile taken at the $\overline{\Gamma }$-point (green spectrum) allows us to identify electronic states with antinodes at the centre of the BZ, which we label as *N* = 1–4. The simulated LDOS for these four selected states are shown in Fig. 5c. While the *N* = 1 mode exhibits localization and spread of the LDOS intensity from the center of the QD similar to the ground state *n* = 1 for coronene, higher order modes *N*>1 exhibit additional antinodes at the boundary and interior of the QD with the number of these additional antinodes increasing with the mode number, remarkably reassembling, as anticipated, the quasiparticle standing wave interference patterns reported for metallic quantum corrals^33,44^.

### Coupled graphene quantum dots: artificial atoms and quantum stadium

**Figure 6:**
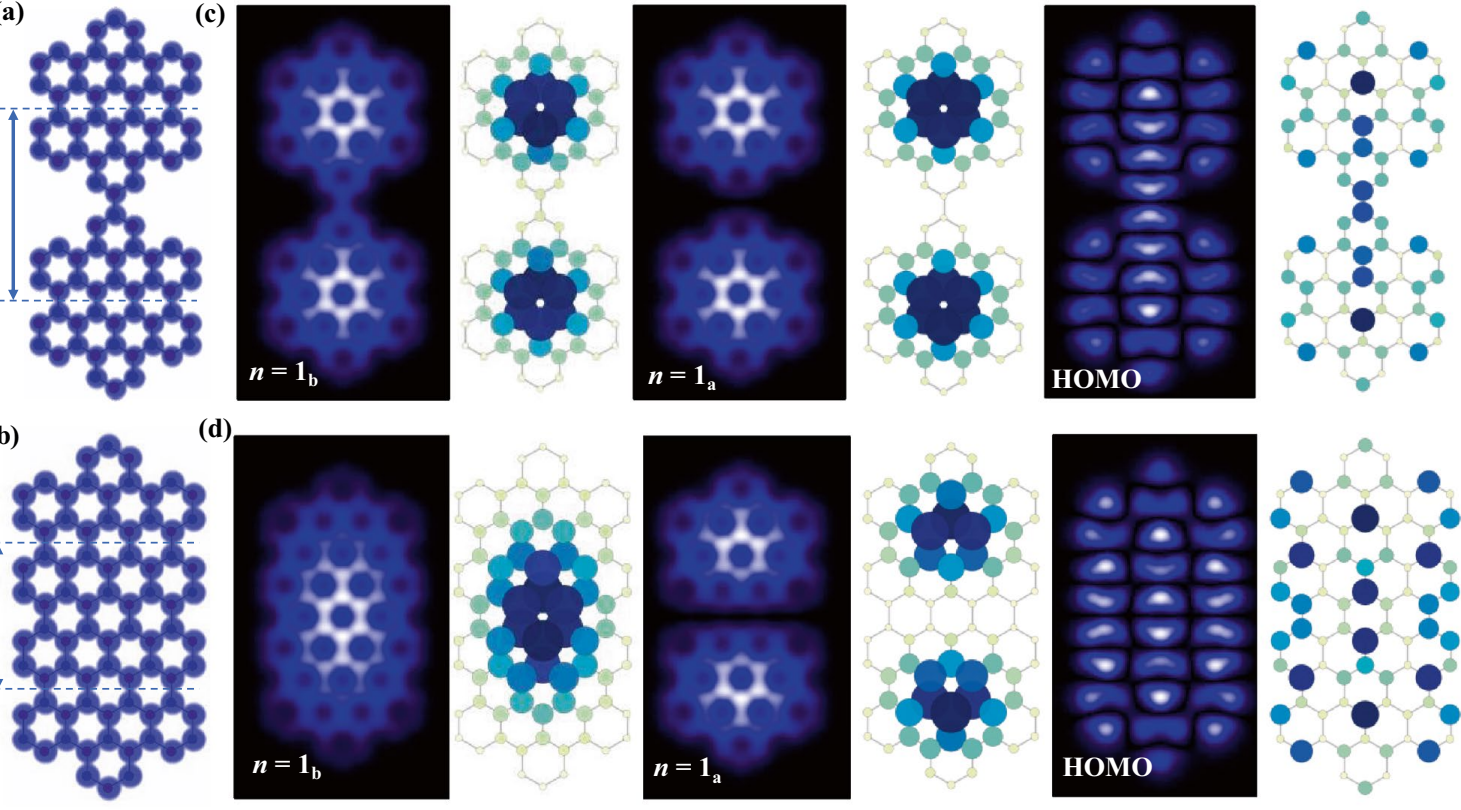
Graphene artificial atoms and quantum stadium. (**a**, **b**) EPWE potential landscapes and the corresponding TB models superimposed for two coupled GQDs with separation 9*a* (**a**) and 6*a* (**b**) defining weak and strong coupling regimes, respectively. (**c**, **d**) EPWE and TB LDOS simulations for bonding (*n* = 1_b_) (left) and antibonding (*n* = 1_a_) (middle) states as well as the HOMO (right) for weakly (**c**) and strongly (**d**) coupled GQDs.

The analogy here discussed between graphene and metallic QDs remains applicable even for more complex nanostructures such as quantum stadiums^62,63^ and coupled QDs, experimentally reported for CO-based nanostrucutres tip-assembled onto metallic substrate^64^, where bonding and antibonding states in these metallic artificial atoms/molecules mimicking the electronic structure of diatomic molecules were identified. In Fig. 6a, b we present EPWE potential landscapes and the corresponding tight binding (TB) carbon atomic sites for two GQDs, namely hexabenzocoronene, for two coupling regimes; that is weakly (a) and strongly (b) coupled GQDs, with separation of 9*a* and 6*a*, respectively. While the configuration in (a) represents the case of a coupled molecular dimer, the model in (b) is analogous to a graphene quantum stadium. The ground state *n* = 1 of the individual GQD is split upon the weak coupling into bonding *n* = 1_b_ and antibonding *n*=1_a_ states, the wavefunctions of which are simulated using EPWE and TB models and are depicted in Fig. 6c. Both the *n* = 1_b_ (left) and *n* = 1_a_ (middle) states exhibit LDOS localization at the individual QD centers with some weak bonding contribution at the junction position solely for the *n* = 1_b_ state and a clear nodal line for *n* = 1_a_. Since the coupling between the two GQDs is exceptionally weak, akin to isolated GQDs, the simulated HOMO state (right) is practically the same as the individual hexabenzocoronene QD^65^. Similar behavior is expected and has been already measured for the near-Fermi relativistic Dirac electrons due to intravalley scattering in relatively large and coupled graphene QDs^66,67^. By reducing the separation between GQDs from 9*a* to 6*a* (b), we arrive at the situation of a quasi-elliptical GQD similar to quantum stadium patterned on metallic substrates. In such a strong coupling regime, the bonding state *n* = 1_b_ is completely delocalized over the entire structure as a single unit (d, left), being maximized at the elliptical GQD center. Likewise, the HOMO state (d, right) exhibits delocalization over the entire structure as a single molecular unit. The antibonding state *n* = 1_a_(d, middle), on the other hand, reveals high intensity at the individual focal points, i.e., being sensitive to the constituting individual QD centers, with a nodal line at the semi-minor axis of this elliptical corral. The agreement between our EPWE calculations and the simple nearest-neighbour TB model facilitates the exploration of several nanometer large GQD using TB for which confinement of near-Fermi Dirac electrons can be modeled^62,66,67^.

### Large nanoscale graphene quantum dots: intervalley vs. intravalley scattering

**Figure 7:**
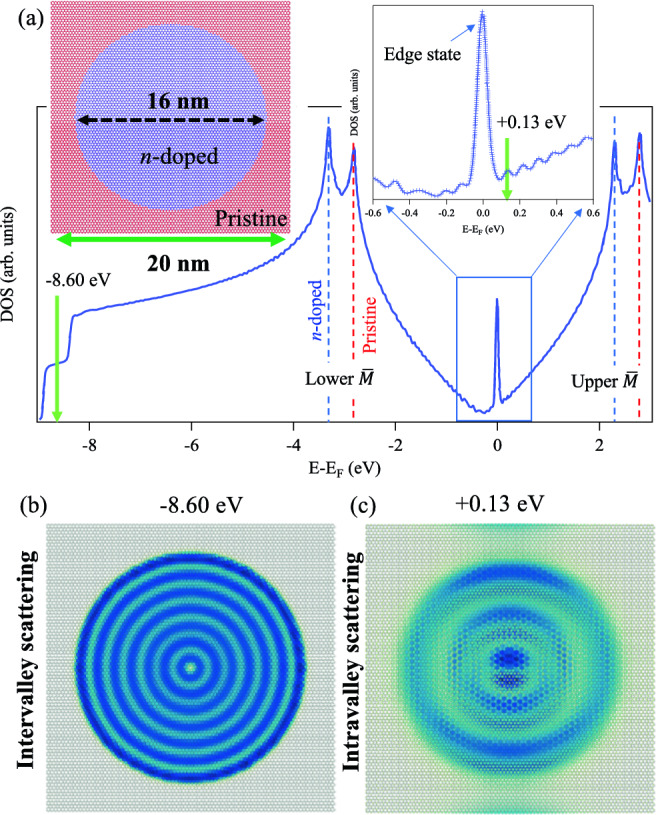
Nanoscale graphene quantum dots: intervalley vs. intravalley scattering. (**a**) TB atomic model for *n*-doped (− 0.5 eV gated) circular GQD of radius *r* = 8 nm embedded in a pristine square graphene sheet (20 $\times$ 20 nm^2^), and the corresponding total DOS calculations. The dashed lines define the lower and upper edges of the $\overline{M}$-point gap, and the inset shows confined Dirac states near the Fermi, where sharp edge state is present. (**b**, **c**) LDOS simulations at the energies indicated by the green arrows in (**a**), i.e., at − 8.6 eV (**b**) and + 0.13 eV (**c**), revealing quasiparticle oscillations due intervalley and intravalley scattering, respectively.

Figure 7 presents TB simulations for large nanoscale GQD. To allow for feasible comparison with experimental measurements, we model a quantum dot with soft (|*V*| = 0.5 eV) confining potential, which can be fabricated experimentally by applying suitable gating to a circular region within an extended graphene sheet^37,61^. The TB model define a circular GQD of size 16 nm, inside which the on-site energy of all carbon atoms is reduced by − 0.5 eV, compared to the surrounding 20 $\times$ 20 nm^2^ square region of pristine graphene.

Given the large size of the system, the total DOS takes the form of extended graphene with the typical near-Fermi V-like shape. However, the presence of *n*-doped (blue) and pristine (red) regions leads to doubly convoluted features around − 8.5 eV, − 3.0 eV, and + 2.5 eV corresponding to the $\overline{\Gamma }$-points as well as the upper and lower edges of the $\overline{M}$-points, respectively (see dashed red and blue lines). Close to the Fermi energy, see zoomed-in inset, an edge state is present due to the finiteness of the system, but importantly the total DOS reveals a number of peaks reminiscence of confined Dirac electrons. To unravel the scattering mechanism behind these near-Fermi features we present, in Fig. 7b, c, LDOS simulations at the energies indicated by the green arrows in (a), that is close to the $\overline{\Gamma }$-point energy (− 8.6 eV) and near-Fermi (+ 0.13 eV). The LDOS taken at − 8.6 eV (b) exhibits the typical quasiparticle interference pattern known for the large metallic quantum corrals^33,35,63^, which originates from the intervalley scattering, i.e, the same as the confined states and standing wave patterns for small and moderate size GQDs just discussed. Similarly, the LDOS taken for the near-Fermi electrons (+ 0.13 eV) exhibits weaker interference patterns with clearly longer periodicity. Such long scale oscillation can not originate from intervalley scattering which possess large scattering wavevector (in the order of $3.4 \text{\AA }^{-1}$) for such low energy states. A plausible origin behind the appearance of such oscillations is the intravalley scattering as reported both theoretically and experimentally in earlier work^3,25,26,36,40,45,49^.

At the end we state that, while the electronic states near the Fermi energy, such as those facilitated by intravalley scattering, can find potential applications in transport and optical devices^3,5,6,68–70^, the high energy confined states in atomic GQD are not appealing in this context, but could impact selective chemical bonding and mediate catalytic processes through their hybridization with other high energy electronic bands. Furthermore, the energy of these states can be brought closer to the Fermi energy in artificial molecular GQDs such as those produced by STM tip-manipulation of on-surface adatoms^18–20^, and can thereby be utilized to modulate electronic properties of materials or induce some functionality via proximity effect^71^.

## Conclusion

In this manuscript we presented detailed EPWE calculations for graphene and lateral metallic quantum dots of different sizes and shapes. We quantitatively demonstrated that the high binding energy $\pi$-electrons in graphene quantum dots exhibit electronic band structures, confined states, and quasiparticle interference patterns analogous to those reported in metallic nanostructures and quantum corrals, which can be readily identified in available tomographic ARPES experiments. This analogy persists even for the frontier orbitals and in the more complex graphene nanostructures, such as coupled GQDs and quantum stadium. The correspondence between graphene and metallic nanostructures here explored is derived from the equivalence of the intervalley scattering in lateral metal and graphene QDs, which for the latter manifests in small and moderate size quantum dots, while larger nanoscale GQD is known to host additional intravalley scattering for Dirac electrons. The present study should have fundamental implications on the understanding and efficient modeling of diverse graphene nanostructures with tailored applications.

## Theoretical methods

### Electron plane wave expansion (EPWE)

We simulate the electronic properties of extended graphene, 2D homogeneous metal, GQDs, and MQDs by solving the one-electron Schrödinger equation for the corresponding 2D potential landscape defined as $V_{\rm s}(\varvec{R}=(x,y))$. For graphene and GQD systems, the carbons atoms are represented by circles where the potential inside and outside are set to $V_0$=0 and $V_1$=22.95 eV, respectively. For extended graphene and the 2D metal, the potential landscape per unit cell is shown in Fig. 1a. For GQD, the corresponding number of carbon atoms is defined, as depicted in Fig. Fig. 2d, 5a and 6a, b, and they are decoupled in the *x*- and *y*-directions by separating them with $\sim$ 10 Å gaps to mimic a finite QD system. MQDs are explored in the same fashion, except that the potential inside the QDs $V_0$ is set to zero. Solving the Schrödinger equation below, for the eignvalues *E* and eignfunctions $\psi (\varvec{R})$, we can extract the band structure, LDOS, and photoemission intensity as detailed in^21^.

1 $$\begin{aligned} -\frac{\hbar ^2}{2m_{\rm eff}}\nabla ^2\psi (\varvec{R})+V_{\rm s}(\varvec{R})\psi (\varvec{R})=E\psi (\varvec{R}), \end{aligned}$$

The reference energy for graphene and GQD is chosen to set the $\overline{K}$-point of extended graphene at the Fermi energy, while for the metallic systems it was adjusted to have the same $\overline{\Gamma }$-point energy as graphene. For a proper comparison between graphene and 2D metal, the effective mass was slightly enlarged for the later, ($m_{\text {eff}} = 1.165 \, m_e$). A number of reciprocal lattice vectors spanning a distance $g_{\text {max}} = 15$ from the reciprocal lattice origin is used for the extended systems, while larger values of $g_{\text {max}}$ ($\ge 35$) are required to achieve decent convergence for all GQD and MQD systems.

### Tight binding (TB)

We used the open-source Pybinding package^72^ (https://docs.pybinding.site/en/stable/) to simulate the total and local density of states for a nanoscale large GQD, Fig. 7. We define a 20$\times$20 nm^2^ free-standing graphene sheet with on-site energy $\epsilon _0$ and first-nearest neighbour hopping parameter (*t*) of 0 and − 2.8 eV, respectively. We then set $\epsilon _0$ = − 0.5 eV for all carbon atoms enclosed within a circle of radius *r* = 8 nm, from the center of the graphene sheet, thereby defining a *n*-doped GQD.

### Supplementary Information

Supplementary Movie 1.

Supplementary Movie 2.

## Data Availability

The data presented in this study are available on request from the corresponding authors.
